# Loss of Proprotein Convertase Furin in Mammary Gland Impairs proIGF1R and proIR Processing and Suppresses Tumorigenesis in Triple Negative Breast Cancer

**DOI:** 10.3390/cancers12092686

**Published:** 2020-09-20

**Authors:** Zongsheng He, Abdel-Majid Khatib, John W. M. Creemers

**Affiliations:** 1 Laboratory of Biochemical Neuroendocrinology, Department of Human Genetics, KU Leuven (Katholieke Universiteit Leuven), 3000 Leuven, Belgium; zongsheng.he@kuleuven.be; 2 INSERM, LAMC, UMR 1029, Allée Geoffroy St Hilaire, 33615 Pessac, France; 3 Digestive group, Institut Bergonié, 33000 Bordeaux, France

**Keywords:** triple negative breast cancer, Furin, AKT, ERK1/2

## Abstract

**Simple Summary:**

Triple-negative breast cancer (TNBC) is known to have a poor prognosis and limited treatment options. The aim of the current study is to evaluate the role of Furin, a proprotein convertase involved in the activation of wide range of protein precursors in TNBC progression. The generation of a TNBC mouse model lacking Furin specifically in the mammary gland confirmed that Furin is implicated in TNBC tumor progression and the derived lung metastasis. Further analysis revealed that the proteolytic activation of proIGF1R and proIR receptors, two substrates of Furin involved in TNBC were inhibited in these mice and was associated with reduced AKT and ERK1/2 expression and phosphorylation. In addition, Furin is frequently overexpressed in TNBC tumors and correlates with poor patient prognosis, suggesting the use of Furin inhibition as a potential adjunct therapy in TNBC.

**Abstract:**

In triple negative breast cancer (TNBC) cell lines, the proprotein convertase Furin cleaves and then activates several protein precursors involved in oncogenesis. However, the in vivo role of Furin in the mammary gland and how mammary gland-specific Furin knockout specifically influences tumor initiation and progression of TNBC is unknown. Here, we report that Furin is frequently overexpressed in TNBC tumors and this correlates with poor prognosis in patients with TNBC tumors. In a whey acidic protein (WAP)-induced mammary epithelial cell-specific Furin knockout mouse model, mice show normal mammary development. However, loss of Furin in mammary glands inhibits primary tumor growth and lung metastasis in an oncogene-induced TNBC mouse model. Further analysis of TNBC mice lacking Furin revealed repressed maturation of the Furin substrates proIGF1R and proIR that are associated with reduced expression and activation of their downstream effectors PI3K/AKT and MAPK/ERK1/2. In addition, these tissues showed enhanced apoptotic signaling. In conclusion, our findings reveal that upregulated Furin expression reflects the poor prognosis of TNBC patients and highlights the therapeutic potential of inhibiting Furin in TNBC tumors.

## 1. Introduction

Breast cancer is a heterogeneous disease with variable histological characteristics, clinical outcomes and treatment responses. Based on the expression status of the estrogen receptor (ER), progesterone receptor (PR) and human epidermal growth factor receptor 2 (HER2), breast cancers are classified as four subtypes including Luminal A, Luminal B, HER2 and triple-negative breast cancer (TNBC) [1,2]. For Luminal A/B or HER2, treatment options aim to inhibit the estrogen pathway or target amplified HER2, respectively, which is effective for treating patients whose tumors express these targets. However, TNBC tumors do not express ER, PR and HER2, and therefore effective treatments for TNBC are limited.

Furin, together with six other members (PC1/3, PC2, PC4, PACE4, PC5/6 and PC7), constitute the proprotein convertase (PC) family that is responsible for the cleavage and activation of various protein precursors in cells [3]. Maturation of several of these precursors is required for various processes including tumor initiation, progression and metastasis [4]. Previously, Cheng et al. reported that mRNA expression of Furin in human breast cancer was much higher compared to adjacent breast tissues [5], suggesting that Furin might play an important role in breast cancer. Indeed, several in vitro studies have demonstrated that inhibition of Furin repressed the proliferative, invasive and metastatic potential of various cancer cells, including TNBC cells, by suppressing the cleavage of proproteins including proPDGF-A [6,7] and matrix metalloproteinase 9 [8]. In addition, many other Furin substrates like IGF1R [9,10] and c-MET [11,12] have been reported to maintain the tumorigenic properties of TNBC cells. For example, inhibition of IGF1R represses tumor growth and induces apoptosis in different TNBC cell lines [13]. Inhibition of IGF1R could also enhance the efficiency of PI3K inhibition in multiple TNBC cell lines [14]. Furthermore, Kim et al reported that knockdown of c-MET led to decreased cell proliferation and migration ability in TNBC cell lines [15]. Other studies have reported that well-established Furin substrates, including NOTCH1 [11,16] and TGFβ1 [11,17], influence the oncogenic capabilities of TNBC cells [18,19]. Altogether, these studies indicate that Furin plays an important role in TNBC. 

The promotors of mouse mammary tumor virus (MMTV) and whey acidic protein (WAP) have been used to generate Cre driver lines specifically for mammary tissue [20]. MMTV-Cre is confined to the epithelial cells of secretory cells including the salivary gland and mammary gland. The MMTV-Cre line has been used to inactivate genes in the salivary gland [21] or prostate gland [22]. In addition, the MMTV-Cre line is commonly used to drive the expression of polyoma virus middle T (PyMT) oncoprotein in the mammary gland to generate a spontaneous transgenic breast cancer mouse model [23]. Importantly, several studies have demonstrated that MMTV-PyMT mice on a C57BL/6 background is a representative mouse model for TNBC, since MMTV-PyMT tumors show negative expression of ER, PR and HER2 [24,25]. In contrast, WAP-Cre is detected in epithelial cells of mammary tissue during lactation. Therefore, the WAP-Cre line has been used to specifically inactivate genes in the mammary gland.

In this study, we analyzed the relationship between Furin expression and tumor progression in human TNBC tumors. To investigate the effect of mammary gland-specific Furin deficiency on tumor progression and lung metastasis in TNBC, we established a Furin knockout mouse model using WAP-Cre that we intercrossed with a PyMT-induced TNBC mouse model. 

## 2. Results

### 2.1. Furin Is Highly Overexpressed in TNBC Tumors and Correlates with Poor Prognosis in TNBC Patients

**Figure 1:**
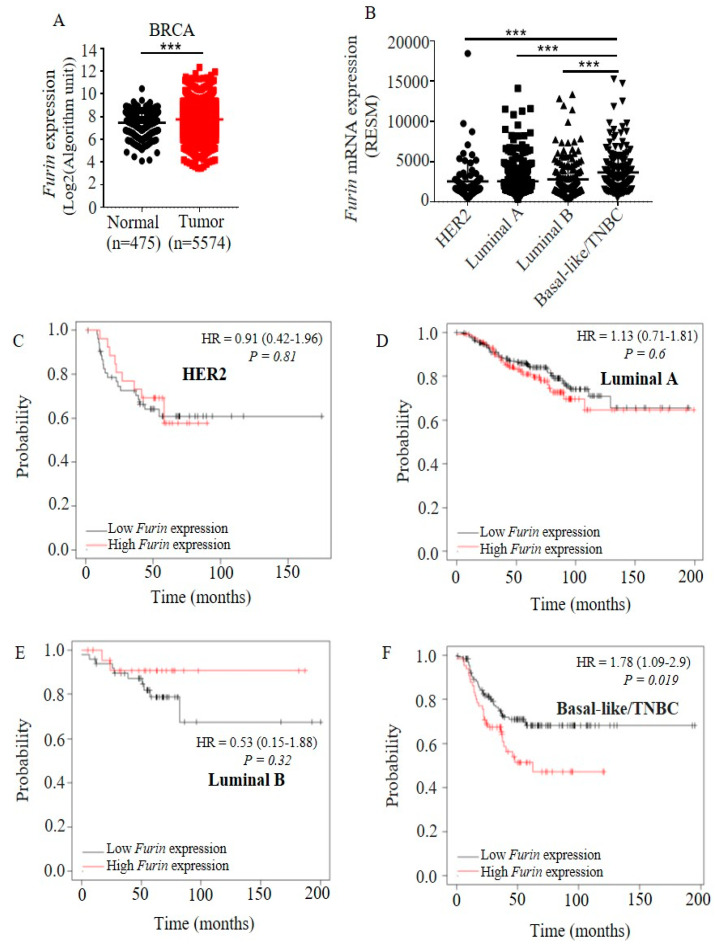
High Furin expression is found in triple-negative breast cancer (TNBC) tumor tissues and correlates with poor prognosis in TNBC patients. (**A**) The expression levels of Furin in all breast cancer cells and matched normal tissue. Each dot represents one sample. Shown is the mean ± SD. (**B**) The expression of Furin in different breast cancer subtypes. Each dot represents one sample. Shown is the mean ± SD. All data are from The Cancer Genome Atlas database. (**C**–**F**) Relapse-free survival curves of breast cancer patients split at the upper tertile of Furin mRNA level. Kaplan–Meier relapse-free survival curves are plotted based on subtypes: (**C**) HER2 (*n* = 79), (**D**) Luminal A (*n* = 352), (**E**) Luminal B (*n* = 76) and (**F**) TNBC (*n* = 198). Patients with tumors with high expression of Furin are shown in red and those with low expression are shown in black. Log-rank p values and hazard ratios (HR, 95% confidence interval in parentheses) are displayed. BRCA: Breast invasive carcinoma. RSEM: RNA sequencing by expectation maximization. HR: Hazard ratio. A two-tailed Student’s t-test was performed for A and B. *** *p* < 0.001.

To identify whether Furin expression is deregulated during breast carcinogenesis, we analyzed the mRNA expression of Furin in human breast cancer tissues and normal breast tissues using the Gent2 online tool (http://gent2.appex.kr/gent2/) [26]. Furin mRNA expression was significantly increased in tumor tissues compared to normal breast tissues (Figure 1A). We further analyzed the Furin expression in different breast cancer subtypes. TNBC tumor tissues showed higher expression levels of Furin compared to other subtypes including HER2, Luminal A and Luminal B (Figure 1B), indicating that upregulated Furin expression plays a more important role in TNBC compared to the other three subtypes. Next, we analyzed the role of Furin expression in tumor progression in the different breast cancer subtypes via the online tool Kaplan–Meier Plotter (https://kmplot.com/analysis/) [27]. 

Of the four breast cancer subtypes, we did not find a significant correlation between Furin expression and tumor prognosis in HER2, Luminal A and Luminal B tumor samples (Figure 1C–E). In contrast, we observed that lower Furin expression was correlated with a significant improvement in relapse free survival (RFS) in 198 TNBC samples (Figure 1F). Furthermore, we also observed that the high Furin expression group in TNBC had the worst tumor prognosis (50%) of all four types of breast cancer (Figure 1F). These data reveal that upregulated Furin expression is linked to tumor progression in TNBC.

### 2.2. Targeted Inactivation of Furin in the Mammary Gland via WAP-Cre Induces Normal Mammary Development

**Figure 2:**
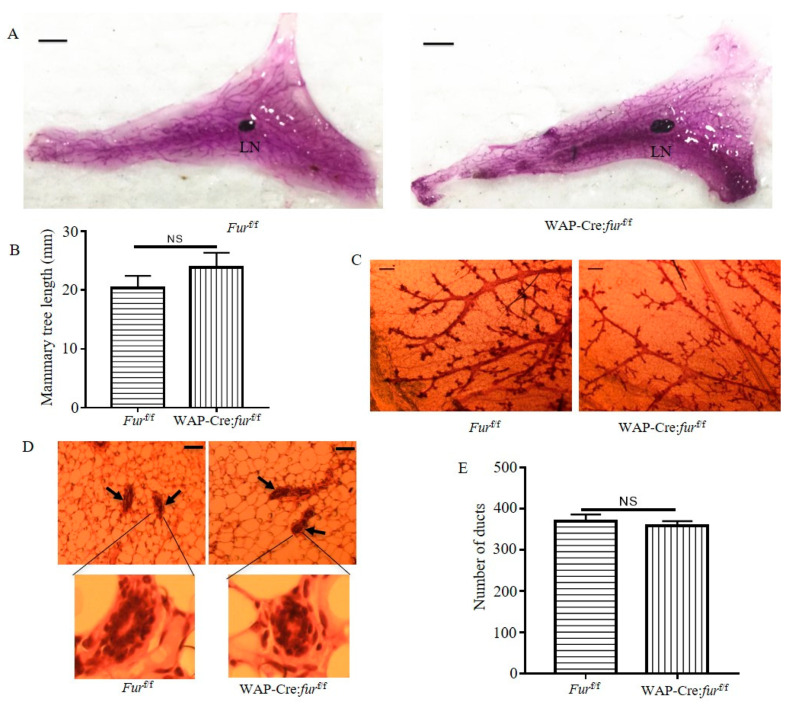

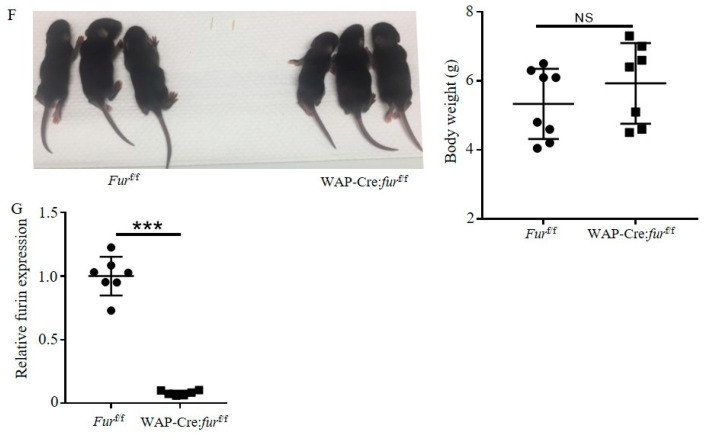
Normal mammary development in mice with Furin deficiency in the mammary glands. (**A**) Representative photos of mammary gland whole-mount carmine alum staining for multiparous furf/f or WAP-Cre:furf/f female mice. Scale bar: 5 mm. LN: lymph node. (**B**) Quantification of length of the longest mammary tree past the lymph node in the whole mammary gland. *n* = 3 for each group. (**C**) Representative photos of whole-mount carmine alum staining of ductal epithelium from multiparous furf/f or WAP-Cre:furf/f female mice. Scale bar: 200 µm. (**D**) Representative H&E staining of mammary glands from multiparous furf/f or WAP-Cre:furf/f female mice. Scale bars: 50 μm. *n* = 3 for each group. (**E**) The number of ducts in H&E stained sections of mammary glands from multiparous furf/f or WAP-Cre:furf/f female mice. (**F**) The 10-day-old pups fed by a multiparous fur f/f female or WAP-Cre:furf/f female mouse (left panel). The body weight of 2 litters of 10-day-old pups fed by a multiparous furf/f female or WAP-Cre:furf/f female mouse (right panel). Each dot represents one pup. (**G**) The mRNA expression of Furin in mammary glands from multiparous furf/f female or WAP-Cre:furf/f female mice. A two-tailed Student’s t-test was performed for **B**, **E**, **F** and **G**. NS: not significant, *** *p* < 0.001.

To evaluate the role of Furin in mammary development, we established a mammary gland-specific Furin knockout mouse model. We crossed WAP-Cre mice with Furin flox/flox (furf/f) mice to inactivate Furin in the mammary gland, yielding WAP-Cre: furf/f mice. All WAP-Cre: furf/f mice were phenotypically normal at birth, developed normally and were fertile. After multiple rounds of pregnancy, these multiparous female mice showed normal mammary gland development (Figure 2A). We further analyzed mammary tree elongation by measuring the mammary tree length related to the lymph node. Quantitative analysis showed that the relative duct length in whole mounts from multiparous WAP-Cre:furf/f female mice was similar to multiparous furf/f female mice (Figure 2B). Furthermore, we observed similar branching patterns between the two groups (Figure 2C). We then analyzed the ductal structures in hematoxylin and eosin-staining of tissue sections. This staining revealed that the ductal structures in multiparous WAP-Cre:furf/f female mice mammary glands were evenly distributed along adipocytes in the mammary gland and were not different from those of multiparous furf/f female mice (Figure 2D). Moreover, the number of mammary ducts was not different between the two groups (Figure 2E). In addition, WAP-Cre:furf/f female mice produced sufficient milk to nurse their litters properly, and we did not observe growth retardation and morbidity in their offspring (Figure 2F). These pups had a similar body weight at about 10 days compared to pups of furf/f multiparous female mice (Figure 2F). 

Next, we analyzed Furin expression in mammary glands isolated from furf/f or WAP-Cre:furf/f multiparous female mice. qRT-PCR data indicated that *Furin* mRNA expression was reduced more than 50-fold in multiparous WAP-Cre:furf/f female mice. Taken together, these data reveal that loss of Furin in mammary epithelial cells has no apparent effect on the mammary development despite near-complete inactivation (Figure 2G).

### 2.3. Inactivation of Furin in the Mammary Gland Inhibits Tumor Growth and Lung Metastasis in PyMT Induced TNBC Mice

**Figure 3:**
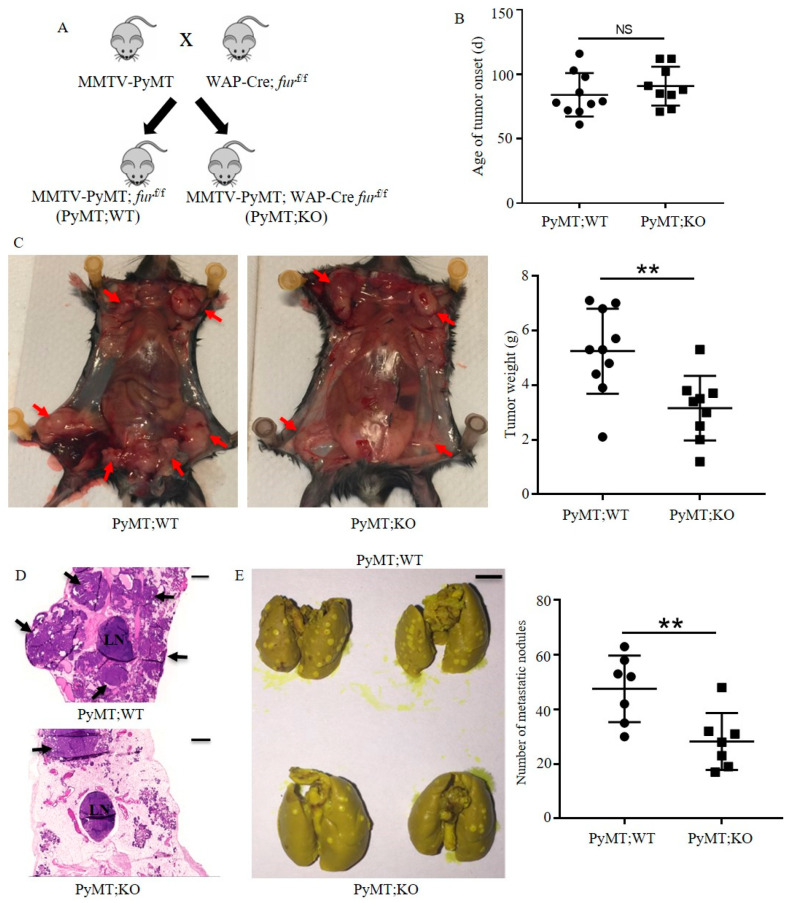
Inactivation of Furin in the mammary gland of TNBC mice inhibits tumor burden and lung metastasis. (**A**) Breeding scheme of Furin knockout in mammary epithelial cells in PyMT-driven TNBC mouse model. (**B**) Age of tumor onset in multiparous PyMT;WT and PyMT;KO female mice. (**C**) Mammary tumor weight of multiparous PyMT;WT and PyMT;KO female mice at the endpoint. Representative photos of tumor burden in multiparous PyMT;WT and PyMT;KO female mice (arrows indicate mammary tumor in left panel); weight of the mammary tumors of multiparous PyMT;WT and PyMT;KO female mice (right panel). (**D**) H&E staining of tumor samples described in C. Arrows represent high-grade carcinoma area in mammary gland from multiparous PyMT;WT and PyMT;KO female mice. Scale bars: 50 µm. (**E**) Lung metastasis of multiparous PyMT;WT and PyMT;KO female mice. Representative photographs of lung metastasis (left panel). Yellow dots represent metastatic nodules. Scale bars: 5 mm. The number of metastatic nodules in the lung of multiparous PyMT;WT and PyMT;KO female mice (right panel). LN: lymph node. A two-tailed Student’s t-test was performed for **B**, **C** and **E**. NS: not significant, ** *p* < 0.01.

Since MMTV-PyMT phenotypes of the C57BL/6 strain showed absence of ER, PR and HER2 in PyMT tumor cells [24,25], we used this strain to determine the effect of mammary gland-specific Furin knockout on tumor progression of TNBC. To this end, we crossed MMTV-PyMT mice to WAP-Cre: furf/f to disrupt mammary epithelium-specific Furin in MMTV-PyMT mice (Figure 3A). Multiparous female mice were monitored for the development of mammary neoplasms. Tumor onset in multiparous MMTV-PyMT mice was relatively varied, with most tumors arising between 71 and 116 days (Figure 3B). The average period of tumor onset was similar between multiparous MMTV-PyMT; WAP-Cre furf/f (PyMT;KO) and MMTV-PyMT;furf/f (PyMT;WT) mice (Figure 3B). However, we observed that PyMT;KO mice had significantly smaller tumor mass than PyMT;WT mice (Figure 3C). Histopathological analysis of mammary tumor also showed that the degree of high-grade carcinoma areas in PyMT;KO mice was lower than those of PyMT;WT mice (Figure 3D). These data indicate that loss of Furin in mammary epithelial cells does not contribute to inhibiting tumor onset, but it inhibits mammary tumor growth in PyMT-driven TNBC mouse model.

MMTV-PyMT mice always show lung metastasis at late stages of mammary carcinogenesis [23]. To explore the effect of mammary gland-specific Furin knockout on lung metastasis in these mice, we analyzed the total number of metastatic nodules in harvested lung tissue of PyMT;KO mice and PyMT;WT mice by means of Bouin’s staining. We observed that PyMT;WT mice showed multiple metastatic nodules varying from 30 to 63 (Figure 3E). In contrast, PyMT;KO mice had a lower number of metastatic nodules varying from 17 to 48, indicating that inhibited tumor growth in the mammary gland lowered lung metastasis. Taken together, these data indicate that disruption of Furin in mammary epithelial cells suppresses the dissemination of mammary tumor cells in PyMT-driven TNBC mice.

### 2.4. Furin Inactivation in the Mammary Gland of TNBC Mice Inhibits the Proteolytic Maturation of proIGF1R and proIR and Downregulates PI3K/AKT and MAPK/ERK1/2 Expression and Activity 

**Figure 4:**
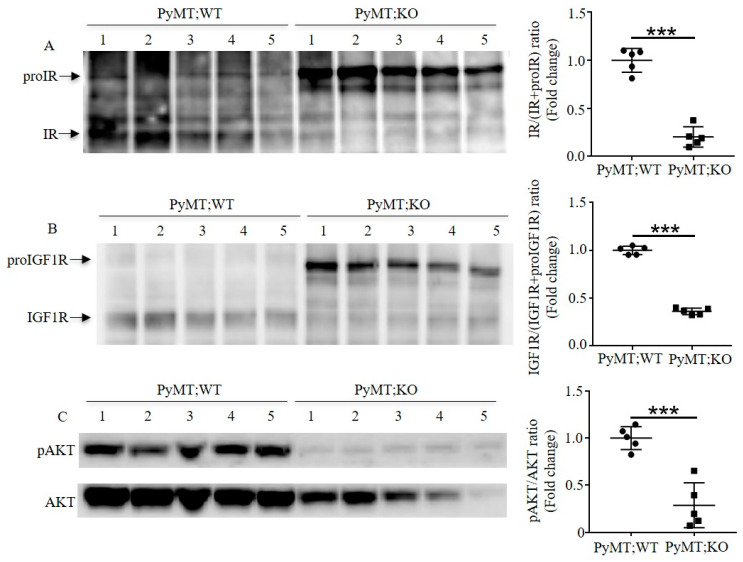

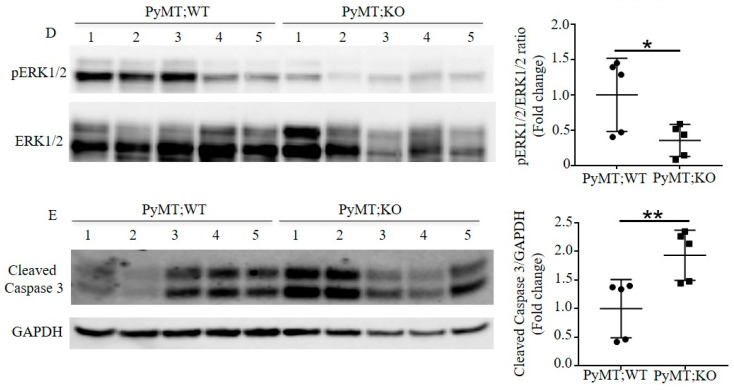
Inactivation of Furin in the mammary gland enhances apoptosis by impairing maturation of proIGF1R and proIR in TNBC mice. (**A**) Immunoblotting analysis of proIR and IR in tumor samples of multiparous PyMT;WT and PyMT;KO mice. Representative photo of protein levels of proIR and IR in tumor samples (left panel); quantification of IR/IR+proIR ratio in indicated tumor samples (right panel). (**B**) Immunoblotting analysis of proIGF1R and IGF1R in tumor samples of multiparous PyMT;WT and PyMT;KO mice. The protein levels in tumor samples are shown in the left panel; the quantification of IGF1R/IGF1R+proIGF1R ratio in indicated tumor samples in the right panel. (**C**) Inactivation of Furin in mammary epithelial cells reduces PI3K/AKT expression and signaling in TNBC tumor samples. Immunoblotting analysis of phosphorylated AKT and total AKT in indicated TNBC tumor samples (left panel). The quantification of pAKT and AKT ratio is indicated in the right panel. (**D**) Inactivation of Furin in mammary epithelial cells reduces MAPK/ERK1/2 expression and activity in TNBC tumor samples. Immunoblotting analysis of phosphorylated ERK1/2 and ERK1/2 in TNBC tumor samples is shown in the left panel and the quantification of pERK1/2 and ERK1/2 ratio is shown in the right panel. (**E**) Immunoblotting analysis of cleaved caspase 3 in TNBC tumor samples. Protein levels of cleaved caspase 3 in TNBC tumor samples (left panel) and quantitative analysis of cleaved caspase 3/GAPDH ratio in indicated tumor samples of TNBC mice (right panel). A two-tailed Student’s t-test was performed for A–E. * *p* < 0.05. ** *p* < 0.01. *** *p* < 0.001.

To investigate how mammary gland-specific Furin influences the tumor progression in TNBC mice, we analyzed Furin substrates in mice-TNBC developed tumors via immunoblotting (Figure 4 and Figure S1). As illustrated in Figure 4A,B, Furin inactivation in mammary epithelial cells impaired proIGF1R and proIR processing and led to reduced amounts of cleaved IGF1R and IR in tumor mass of PyMT;KO mice (Figure 4A,B). Next, we assessed how decreased levels of IGF1R and IR influenced oncogenic signaling pathways in TNBC mice. Therefore, we examined the activation of PI3K/AKT and MAPK/ERK1/2 signaling mediated by IR and IGF1R in TNBC tumors. Immunoblotting analysis revealed reduced total AKT expression in tumors of PyMT;KO mice compared to PyMT;WT mice tumors (Figure 4C). Further analysis also revealed reduced AKT phosphorylation in these tumors. Indeed, quantitative analysis of pAKT/AKT ratio in both groups indicated that decreased levels of mature IGF1R and IR forms is associated with reduced activity of PI3K/AKT signaling in TNBC tumors. Furthermore, we found that there was also a decrease in the expression and phosphorylation of ERK1/2 level in tumor samples of PyMT;KO mice, compared to PyMT;WT mice (Figure 4D). These data indicate that Furin deficiency impairs proIGF1R and proIR processing that is associated with reduction in their downstream pathways PI3K/AKT and MAPK/ERK1/2 expression and activity in TNBC tumor cells. 

Previously, inhibition of Furin activity has been reported to affect the survival of cancer cells in vitro [7,28]. To directly assess how downregulation of PI3K/AKT and MAPK/ERK1/2 signaling represses the tumor growth in vivo in TNBC mice, we evaluated the protein levels of cleaved caspase 3 in tumor samples of both groups, which have been previously reported to be regulated by Furin in apoptotic cells [29]. We found that cleaved caspase 3 was significantly upregulated in tumor samples of PyMT;KO mice compared to PyMT;WT mice (Figure 4E), indicating that more tumor cells had undergone apoptosis in tumor samples of PyMT;KO mice. This is consistent with the lower activity of PI3K/AKT and MAPK/ERK1/2 signaling in tumor samples of PyMT;KO mice. The increased number of apoptotic tumor cells explains, at least in part, the lower probability of lung metastasis in PyMT;KO mice. All in all, these data suggest that loss of Furin in mammary epithelial cells inhibited tumor progression and lung metastasis by interfering with maturation of proIGF1R and proIR in PyMT-driven TNBC mice.

## 3. Discussion

**Figure 5:**
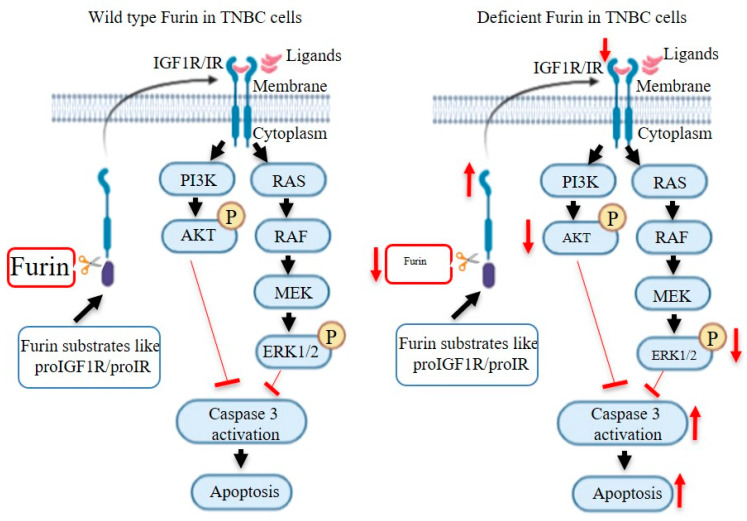
Schematic representation of the effect of Furin deficiency on apoptosis in TNBC cells. After cleavage by Furin, IGF1R/IR can be stimulated by their ligands like IGF1 and insulin. Activated IGF1R/IR results in signaling via two pathways: PI3K/AKT and RAS/ERK1/2. When AKT and ERK1/2 are phosphorylated, caspase 3 activity is inhibited, resulting in reduced cell apoptosis. In Furin-deficient TNBC cells, proIGF1R and proIR will accumulate. Decreased IGF1R/IR leads to reduced phosphorylation of AKT and ERK1/2 after stimulation with IGF1 and insulin. Therefore, the inhibitory effects of pAKT and pERK1/2 on caspase 3 activity are decreased, which enhances apoptosis in TNBC cells. Furin is also required for the activation of other substrates involved in the expression and activity of PI3K/AKT and RAS/ERK1/2 pathways.

In this study, we report that human TNBC tumors show upregulated Furin expression, which correlates with a worse clinical outcome. Disruption of Furin in mouse mammary epithelial cells did not interfere with the development and function of mammary gland. In a PyMT-induced TNBC mouse model, mammary epithelial cell-specific Furin knockout resulted in a reduced tumor load and lung metastasis. This is, at least in part, the result of impaired processing of proIGF1R/proIR and the associated reduced AKT/ERK1/2 expression and signaling leading to increased apoptosis in tumor cells (Figure 5). 

Although mammary gland development is regulated by the sex steroids progesterone and estrogen, the important role of other growth factors like TGFβ1and IGF1 has been confirmed. For example, TGFβ1 is implicated in regulating mammary morphogenesis and secretory function by influencing epithelial survival, proliferation and its extracellular matrix [30]. In addition, Kleinberg et al. reported that IGF1 knockout mice showed impaired mammary development [31]. In contrast, we observed no abnormal phenotypes including mammary branch morphogenesis and mammary tree length in Furin knockout mice (Figure 2A,C). Furthermore, we also found that the pups from multiparous WAP-Cre: furf/f mice grow normally (Figure 2F), indicating that Furin deficiency does not influence milk production and secretion of mammary glands. This observation suggests that the residual production of IGF1R, IR and probably other factors is enough for normal mammary development and function but insufficient to support tumor progression. Alternatively, it is possible that IGF1 or TGFβ1 play important roles in mammary gland development in an endocrine manner. In case of an endocrine role, the production of IGF1 or TGFβ1 by other cell types in which Furin is not inactivated, might have compensated for the loss of mammary epithelial cell produced IGF1 and TGF β1. Indeed, using Furin knockout mice to study the specific role of Furin during development revealed that Furin null mice die between e10.5 and e11.5 due to severe ventral closure defects, indicating the vital, non-redundant functions of Furin at early developmental stages [32]. In contrast, we observed a high degree of redundancy of Furin in adult liver [33]. Interestingly, the breast is the only organ to develop mostly after birth [34], suggesting that the absence of Furin can be compensated for by other PCs to secure the normal development and function of the mammary glands.

The tumor progression induced by PyMT includes multiple stages: hyperplasia, adenoma, and early and late carcinoma [23]. Different tumor suppressor genes or oncogenes are dynamically involved in tumor progression at different stages [35]. Since Furin-deficient mammary glands show a normal phenotype, it is possible that Furin substrates have a limited effect on tumor growth in the early stage. As a result, PyMT;KO mice show a similar tumor onset period compared to PyMT;WT mice. As the tumor develops, more oncogenic pathways like MAPK and PI3K signaling pathways become involved in the tumorigenesis. Therefore, many Furin substrates like IGF1R and IR influence tumor progression at a late stage. As our data show, proIGF1R and proIR processing is impaired (Figure 4A,B), which leads to repressed tumor growth in PyMT;KO mice (Figure 3C). Indeed, the importance of Furin processing of various precursors may explain the anti-tumorigenic and anti-metastatic effects of Furin silencing in PyMT;KO mice. Defects in IGF1R or IR expression and/or activation inhibit tumorigenicity, and cause substantial apoptosis in vitro and in vivo [36,37]. This anti-oncogenic effect of IGF1R inactivation was reported to involve the modulation of the levels and activation of various effectors required for tumor growth and survival including AKT and ERK1/2 [38]. While the effect of Furin substrate cleavage inhibition is well-known to prevent their ability to mediate the activation of various signaling pathways including AKT and ERK1/2 phosphorylation [9,28], in our TNBC model the repression of Furin reduced not only AKT and ERK1/2 activation but also their expression levels. The mechanism linking Furin to AKT and ERK1/2 expression is presently not clear, but several mechanisms can be postulated. The ability of Furin to activate protein precursors involved in the expression of AKT and ERK1/2, such as Dispatched [39] may be a contributing factor. Dispatched is essential for the release of Hedgehog proteins from the producing cells and Hedgehog-GLI1 was reported to contribute to the survival of cancer cells by promoting transcription of AKT genes by its direct binding in the AKT promoter region [40]. Therefore, inhibition of Dispatched cleavage may directly result in reduced AKT expression. Alternatively, the observed reduction in AKT and ERK1/2 proteins in PyMT;KO tumors could be directly due to their induced degradation in the absence of activated proteins required for their stability, as previously reported for VEGF receptor activation that prevents AKT protein degradation [41]. Indeed, VEGF receptor tyrosine kinase inhibition resulted in decreased AKT protein levels and cell stimulation with VEGF in the presence of VEGF receptor inhibitors rescued AKT stability [41]. Interestingly, the activation of VEGF receptor was also reported to be dependent on VEGF-C cleavage by Furin and other PCs [32]. Thereby, in our model, repression of Furin may generate inactive VEGF-C ligand unable to mediate VEGF receptor activation that in turn results in AKT protein degradation in PyMT;KO tumors. 

The observed enhanced apoptosis in PyMT;KO mice can be explained by Furin repression-mediated blockade of an autocrine/paracrine mechanism involved in cell survival. Since repression of Furin inhibited the processing of proIGF1R and proIR and possibly its ligand proIGF1 [42], reported to be expressed by TNBC, it is likely to abrogate their autocrine/paracrine protective effects as observed in PyMT;KO mice tumors. This abrogated effect is associated with reduced AKT and ERK1/2 activation and expression.

It is well documented that TNBC is also driven by other signaling pathways like androgen receptor (AR) signaling, PI3K signaling and MAPK signaling [43,44,45]. Targeting these pathways is therefore a potential way to inhibit TNBC. The application of AR inhibitors shows clinical benefits in TNBC patients with AR positive tumors [46,47]. In addition, inhibition of MAPK and PI3K signaling-associated proteins like IGF1R also suppresses the tumorigenic properties of TNBC cell lines [48,49].

Furin has been implicated in regulating multiple oncogenic pathways like MAPK and PI3K signaling [11,50]. Our finding here supports the idea that inhibition of MAPK and PI3K signaling by interfering with maturation of proIGF1R and proIR contributes to inhibiting tumor growth by enhancing apoptosis in the PyMT-induced TNBC mouse model (Figure 4C,D). It is not surprising that the reduced tumor growth lowers the probability of lung metastasis in PyMT;KO mice (Figure 3E). It should be noted that other potential Furin substrates that were not studied here, like NOTCH1 and c-MET, might also be involved in the malignancy of TNBC cells [51,52]. 

Recently, immunological interventions have been explored as a promising strategy in treating TNBC, since TNBC is highly enriched in tumor-infiltrating lymphocytes. Clinical trials have showed the therapeutic potential of immunotherapy in TNBC patients. Furin also plays an important role in regulating the immunological system by regulating its substrates. Tomé et al. demonstrated that inhibition of Furin repressed the PD-1 expression in immune cells by blocking proteolytic maturation of the NOTCH precursor and enhanced tumor clearance [53]. We have recently shown that knockout of Furin in T cells can suppress mammary tumorigenesis in PyMT-induced TNBC mice [54]. This study further demonstrates that targeting Furin in mammary gland inhibits mammary carcinogenesis in PyMT-induced TNBC mice. 

In the absence of available, efficient targeted therapy for TNBC, the standard of care for this disease is mainly chemotherapy. However, although TNBC tumors usually show better responses to chemotherapy compared to the other breast cancer subtypes, incomplete responses constitute up to 80% of TNBC patients due to significant heterogeneity within these tumors [55]. Therefore, the development of combinatorial treatments to treat TNBC is critical. In this respect, the oncogenic properties of Furin in TNBC make it a new potential target in various combination therapies using different experimental models prior to clinical trials. 

## 4. Materials and Methods

### 4.1. Mouse Model

Furf/f mice were generated in our lab as previously described [33]. WAP-Cre (on a FVD/N background) mice were from Prof. Jos Jonkers (Netherlands Cancer Institute, Amsterdam, The Netherlands). MMTV-PyMT (on a FVD/N background) mice were from The Jackson Laboratory (Bar Harbor, ME, USA). To generate WAP-Cre and MMTV-PyMT on a C57BL/6 background, we backcrossed to C57BL/6 mice for 8 generations. WAP-Cre mice were subsequently bred with furf/f mice to produce WAP-cre furf/f. The expression of WAP was induced by successive gestation cycles. Female WAP-cre furf/f mice were bred to male MMTV-PyMT mice to obtain WAP-cre furf/f; MMTV-PyMT (PyMT;KO) mice. Furf/f; MMTV-PyMT (PyMT;WT) mice were generated and used as the control. Primer sequences for PCR genotyping are listed: MMTV forward, 5′-GGAAGCAAGTACTTCACAAGGG-3′; MMTV reverse, 5′-GGAAAGTCACTAGGAGCAGGG-3′; Cre forward, 5′-CCTGTTTTGCACGTTCACCG-3′; Cre reverse, 5′-ATGCTTCTGTCCGTTTGCCG-3′; furf/f forward, 5′-GCTGTATTTATTCCGGAGAC-3′; furf/f reverse, 5′-GTAGTTAGGAGCACATACTG-3′. 

### 4.2. Animal Study

Mammary-tumor free survival was determined by palpation. PyMT;KO and PyMT;WT mice were sacrificed at 20 weeks of age or when they met the institutional euthanasia criteria for tumor size. Whole mammary tumors and lung tissues were collected, weighed, and processed for histopathological analysis. All animal studies were approved by the Animal Housing and Experiment Board of the KU Leuven and performed in compliance with the Belgian Council for Laboratory Animal Science (095/2016).

### 4.3. Bioinformatics Assay

For expression of Furin in human breast cancer tumor samples or paired normal breast tissue, expression data of Furin were extracted from the Gent2 online tool [26]. For Furin expression in HER2, Luminal A/B and basal-like/TNBC tumor samples, RNA sequencing by expectation maximization values of Furin expression were extracted via the cBioportal database [56]. Kaplan–Meier plots of the relationship between relapse-free survival and the expression of Furin in patients with breast cancer subtypes were made by an online tool [27].

### 4.4. Whole-Mount Staining of Mammary Glands 

Mice were sacrificed at the indicated ages. For mammary gland whole-mount staining, abdominal mammary glands were harvested, and the resected tissue was flattened onto a microscope slide and fixed in Carnoy’s fixative (60% ethanol, 30% chloroform, 10% glacial acetic acid) for four hours at room temperature. The tissue mount was then washed in 70% ethanol for 15 min, 35% ethanol for 15 min, 15% ethanol for 15 min and rinsed with distilled water for 15 min. Then the tissue was placed in a carmine alum staining solution overnight. Stained whole mammary glands were then washed in 50% ethanol for 15 min, 70% ethanol for 15 min, 95% ethanol for 15 min, 100% ethanol for 15 min and xylene overnight at room temperature. The stained mammary gland was mounted by Pertex (Histolab, Sverige, Sweden) for long-term storage. The length of the longest mammary tree was quantified as the average distance of straight lines from the lymph node to the terminal ends of the three longest mammary epithelial ducts. 

### 4.5. H&E Staining and Bouin’s Solution Staining

Mammary glands or tumor were isolated and then fixed in 4% formaldehyde solution for at least two days. Next, tissues were paraffin-embedded, sectioned (5 μm), stained with H&E. Slides were imaged by Microscope slide scanner (Axio Scan.Z1, Köln, Germany) and analyzed by Zen 2 software (Carl Zeiss, Köln, Germany). For Bouin’s solution staining, lung was harvested and washed by PBS. Then the lung was fixed by 10 fixed in 10% Bouin’s solution (Sigma, Ronkonkoma, NY, USA). The lung metastatic nodules were examined superficially.

### 4.6. Quantitative RT-PCR 

For gene-expression analysis, total RNA from mammary gland tissue was extracted using the NucleoSpin^®^ RNA kit (Macherey-Nagel, Düren, Germany) according to the manufacturer’s protocol. Reverse-transcription of the RNA was performed using random primers iScript™ cDNA Synthesis Kit (Bio-Rad, Hercules, CA, USA). Real-time PCR and data collection were performed with IQ SYBR Green supermix reagent (Bio-Rad) on a CFX connect instrument (Bio-Rad) and the following primers: Furin forward, 5′- GAGCTGAGATCCTGGTTGCT-3′; Furin reverse, 5′- AGGTTGTGGAAGCCATGC-3′. GAPDH forward, 5′- CCCCAATGTGTCCGTCGTG -3′; GAPDH reverse, 5′- GCCTGCTTCACCACCTTCT -3′. Each sample was run in triplicate and normalized to the housekeeping gene GAPDH. Relative fold changes in gene expression were calculated using the ∆CT method with the equation 2∆∆CT. The results are shown as fold changes compared to the control group average.

### 4.7. Isolation of Tumor Samples and Immunoblotting Analysis

Mammary tumor samples were mechanically chopped into small pieces with scalpels and were then digested with DMEM F12 media with 1.0 mg/mL Liberase TL (Sigma Aldrich, Regensburg, Germany) and 0.25 mg/mL DNase I (Sigma Aldrich) and incubated at 37 °C on a shaker for 1 h. Following incubation, samples were filtered through a 70-μm cell strainer and were spun down at 800 × *g* for 10 min. The top fatty layer was removed. Tumor cells were resuspended in RBC lysis buffer and incubated at room temperature for 5 min to remove red blood cells. The singe cells preparation was then sonicated in cell lysis buffer (Cell Signaling Technology, Danvers, MA, USA) with an EDTA-free protease inhibitor cocktail and phosphatase inhibitors (Roche, Basel, Switzerland). After brief sonication, the tumor lysates were centrifuged, and the supernatants were used as cell lysates. Proteins were resolved on 10% precast gels (Invitrogen, Carlsbad, CA, USA) and transferred to a nitrocellulose membrane. After blocking with 5% non-fat milk in PBS with 0.2% Triton X-100, membranes were incubated with the Furin (Homemade), Insulin receptor (Homemade), IGF1R(Santa Cruz Biotechnology, Dallas, TX, USA), phosphorylated AKT (Cell Signaling Technology, Danvers, MA, USA), AKT (Cell Signaling Technology), phosphorylated ERK1/2 (Cell Signaling Technology), ERK1/2 (Cell Signaling Technology), cleaved caspase 3 (Cell Signaling Technology) or GAPDH (Cell Signaling Technology) antibody, followed by the secondary antibody conjugated with horseradish peroxidase. After washing, the bands were visualized with enhanced chemiluminescence substrate (Perkin Elmer, Waltham, MA, USA) and quantified using Image Quant LAS4000 (GE Healthcare, Princeton, NJ, USA). 

### 4.8. Statistical Analysis

Data are presented as mean ± SD, unless otherwise indicated. Relapse free survival was performed using the Kaplan–Meier method, and the survival of the groups was compared using the log-rank test. A one-way ANOVA was employed when comparing more than two groups. A two-tailed unpaired Student’s t-test was conducted when comparing two groups. *P* < 0.05 was considered statistically significant. Statistics were calculated with Prism (GraphPad 7).

## 5. Conclusions

In conclusion, we demonstrated that mammary epithelial-specific Furin knockout mice develop normal mammary glands. In a PyMT-induced breast cancer mouse model, the disruption of Furin in mammary glands inhibits tumor growth and lung metastasis by inhibiting maturation of proIGF1R and proIR. This study suggests that Furin is a potential target for TNBC treatment and probably other types of cancer.

## Supplementary Materials

The following are available online at https://www.mdpi.com/2072-6694/12/9/2686/s1, Figure S1: Original immunoblotting images used to generate for Figure 4.
