# Supplementary material for: Loss of Proprotein Convertase Furin in Mammary Gland Impairs proIGF1R and proIR Processing and Suppresses Tumorigenesis in Triple Negative Breast Cancer
Source: Cancers (Basel). 2020 Sep 20;12(9):2686. doi: 10.3390/cancers12092686 (PMC7563341; doi:10.3390/cancers12092686)
Supplement: Supplementary file 1 [file cancers-12-02686-s001.pdf]

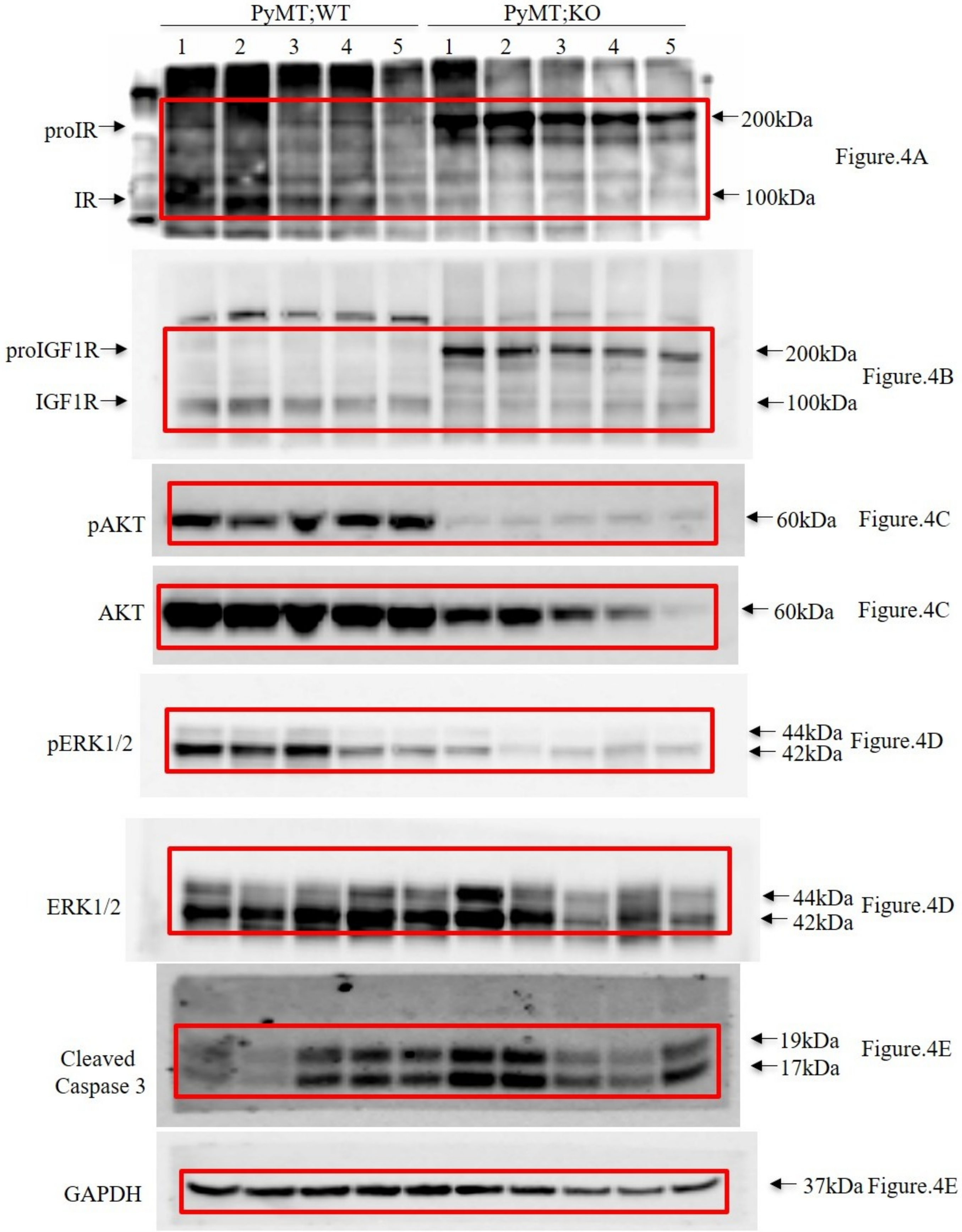

Figure S1. Original immunoblotting images used to generate for Figure 4. Red frame zones are the cropped images within the manuscript. Note: These blots used for pAKT and pERK1/2 were stripped and then blotted for AKT and ERK1/2.
